# Frequency and predictors of the lupus low disease activity state in a multi-national and multi-ethnic cohort

**DOI:** 10.1186/s13075-016-1163-2

**Published:** 2016-11-09

**Authors:** Vera Golder, Rangi Kandane-Rathnayake, Alberta Yik-Bun Hoi, Molla Huq, Worawit Louthrenoo, Yuan An, Zhan Guo Li, Shue Fen Luo, Sargunan Sockalingam, Chak Sing Lau, Alfred Lok Lee, Mo Yin Mok, Aisha Lateef, Kate Franklyn, Susan Morton, Sandra Teresa V. Navarra, Leonid Zamora, Yeong-Jian Wu, Laniyati Hamijoyo, Madelynn Chan, Sean O’Neill, Fiona Goldblatt, Eric Francis Morand, Mandana Nikpour, for the Asia-Pacific Lupus Collaboration

**Affiliations:** 1 Monash University, Melbourne, Australia; 2 The University of Melbourne, Melbourne, Australia; 3 Chiang Mai University Hospital, Chiang Mai, Thailand; 4 People’s Hospital Peking University Health Sciences Center, Beijing, China; 5 Chang Gung Memorial Hospital, Guishan Township, Taiwan; 6 University of Malaya, Kuala Lumpur, Malaysia; 7 University of Hong Kong, Hong Kong, Hong Kong; 8 National University Hospital, Singapore, Singapore; 9 Monash Health, Melbourne, Australia; 10 University of Santo Tomas Hospital, Manila, Philippines; 11 University of Padjadjaran, Bandung, Indonesia; 12 Tan Tock Seng Hospital, Singapore, Singapore; 13 University of New South Wales, Sydney, Australia; 14 Royal Adelaide Hospital, Adelaide, Australia

**Keywords:** Systemic lupus erythematosus, Disease activity, Treatment target, Low disease activity

## Abstract

**Background:**

Systemic lupus erythematosus (SLE) is a chronic heterogeneous disease with considerable burden from disease activity and damage. A novel clinical treatment target in the form of the lupus low disease activity state (LLDAS) has been recently reported, with retrospective validation showing that time spent in LLDAS translates to reduced damage accrual. The objectives of this study were to describe the frequency and identify the predictors of attaining LLDAS in a large multinational cohort of patients with SLE.

**Methods:**

Data were collected at the recruitment visit in patients with SLE enrolled in a longitudinal study in nine countries. Data were analysed cross-sectionally against the recently published definition of LLDAS, and the frequency and characteristics associated with presence of LLDAS were determined. Stepwise multivariable logistic regression was used to determine predictors of LLDAS.

**Results:**

Of the 1846 patients assessed, criteria for LLDAS were met by 44 %. Patients with shorter disease duration were less likely to be in LLDAS (OR 0.31, 95 % CI 0.19–0.49, *p* < 0.001). Likewise, patients with a history of discoid rash (OR 0.66, 95 % CI 0.49–0.89, *p* = 0.006), renal disease (OR 0.60, 95 % CI 0.48–0.75, *p* < 0.001), elevated double stranded DNA (OR 0.65, 95 % CI 0.53–0.81, *p* < 0.001) or hypocomplementaemia (OR 0.52, 95 % CI 0.40–0.67, *p* < 0.001) were less likely to be in LLDAS. When countries were compared, higher national social wealth (OR 1.57, 95 % CI 1.25–1.98, *p* < 0.001) as measured by the gross domestic product per capita was positively associated with LLDAS, but ethnicity was not.

**Conclusion:**

The lupus low disease activity state is observed in less than half of patients with SLE at a single point in time. Disease duration and phenotype, and national social wealth, are predictive of LLDAS.

**Electronic supplementary material:**

The online version of this article (doi:10.1186/s13075-016-1163-2) contains supplementary material, which is available to authorized users.

## Background

Systemic lupus erythematosus (SLE) is a chronic multiorgan autoimmune disease with a broad spectrum of manifestations. Despite global advances in translational research, effective targeted therapies in SLE are lacking [1], and a large proportion of patients are treated with long-term glucocorticoids and non-specific immunosuppressants, which fail to prevent significant morbidity and reduction in life expectancy [2]. The course of SLE is variable, in some cases characterized by periods of relative inactivity punctuated by disease flare, whilst others have persistently active disease [3]. Current instruments used to measure disease activity are complex [4], contributing to mixed results in clinical trials of new targeted therapies [5]. This state of affairs has lead to a call for definitions of treatment target states that can be used in clinical trials and clinical practice [6].

Given that definitions of remission remain under debate [7], and a recently reported stringent definition of remission occurs in only 2 % of patients with SLE [8], using remission as a treatment target is not pragmatic. In other autoimmune diseases, mainly rheumatoid arthritis (RA), achieving a minimally active disease state has been proven to translate into improved patient outcomes [9]. The value of a treatment target for SLE has been recently described in an international consensus statement, in which defining a low disease activity state to use as a treatment target was set as a research agenda [10].

Using consensus methods, the Asia-Pacific Lupus Collaboration has recently developed and retrospectively validated the lupus low disease activity state (LLDAS) definition [11]. The conceptual definition of LLDAS is a state, which if sustained, is associated with good long-term outcomes. The operational definition of LLDAS is fulfilled when all of the following criteria are met: (1) SLE Disease Activity Index (SLEDAI-2 K) ≤4, with no activity in major organ systems (renal, central nervous system (CNS), cardiopulmonary, vasculitis, fever) and no haemolytic anaemia or gastrointestinal activity; (2) no new features of lupus disease activity compared to the previous assessment; (3) a Safety of Estrogens in Lupus Erythematosus National Assessment (SELENA)-SLEDAI physician global assessment (PGA) (scale 0–3) ≤1; (4) a current prednisolone (or equivalent) dose ≤7.5 mg daily; and (5) well-tolerated standard maintenance doses of immunosuppressive drugs and approved biologic agents, excluding investigational drugs. In a retrospective cohort analysis, Franklyn et al. showed that patients who spent greater than 50 % of their disease duration in LLDAS accrued significantly less damage compared to patients who did not [11], suggesting this definition has a role in the identification of treatment responses associated with improved long-term outcomes.

Currently, work is underway to prospectively validate and refine this definition of LLDAS in a large multi-national cohort followed over several years, with the hypothesis that attainment of LLDAS results in less damage accrual. The objective of the current study is to determine the frequency and correlates of LLDAS in a cross-sectional analysis of data collected at recruitment for this study.

## Methods

### Study population

Patients were recruited at 12 centres in nine countries, commencing in May 2013. Each institution obtained ethics approval and written informed patient consent for the study. Patients over the age of 18 years who fulfilled criteria for SLE (either the 1997 American College of Rheumatology (ACR) criteria [12] or the 2012 Systemic Lupus International Collaborating Clinics (SLICC) criteria [13]) were eligible. Data collection took place during the routine ambulatory care of each SLE patient, using a standardized paper or electronic case report form.

### Variables

At recruitment, demographics, disease characteristics and clinical variables were collected from each patient. Demographic variables included gender, ethnicity (self-report based on Australian Standard Classification of Cultural and Ethnic Groups [14]), date of birth, year of definite SLE diagnosis, smoking status, and highest attained education level. Disease manifestations were determined from the ACR classification criteria on an “ever present” basis. Current use and doses of glucocorticoids and immunosuppressive medications were captured for each patient. Disease activity was measured using SLEDAI-2 K [15], and a PGA on a scale of 0–3. Disease flares compared to the previous routine clinical visit were captured using the SLE flare index (SFI) [16]. Irreversible disease damage was captured using the SLICC damage index (SLICC-DI) [17]. Additionally, laboratory results for each patient were obtained within 30 days of the visit, including full blood count, renal function and electrolytes, serum albumin, urine protein/creatinine ratio and microscopy, erythrocyte sedimentation rate, complement 3 and 4, and double stranded DNA (dsDNA) antibody titre.

### Determination of LLDAS

A patient was considered to be in LLDAS if they fulfilled all five predefined criteria [11], with the following modifications. Given the cross-sectional nature of the baseline visit, data collected at recruitment, and hence the absence of data from the previous visit, patients were deemed to be on stable doses of immunosuppressive medications if they did not exceed the maximum recommended dose (Table 3); the criterion for “no new disease activity” was deemed to be met if patients did not meet any SFI criteria.

### Data analysis

Given the young mean age of the patients (Table 1), age at diagnosis ≤30 years was used as a binary variable. Given the likelihood of higher disease activity in the period immediately after diagnosis of SLE [18], disease duration ≤1 year was also used as a binary variable. Patients from different countries were grouped according to gross domestic product (GDP) purchasing power parity per capita [19] in order to account for international differences in socioeconomic status.

**Table 1:** Patient demographics and disease characteristics

	Number (%)^c^ or mean (SD) or median (IQR 25–75) (*n* = 1846 patients)
Country	
Australia	240 (13.00 %)
China	235 (12.73 %)
Hong Kong	190 (10.29 %)
Indonesia	98 (5.31 %)
Malaysia	193 (10.46 %)
Philippines	124 (6.72 %)
Singapore	221 (11.97 %)
Taiwan	295 (15.98 %)
Thailand	250 (13.54 %)
Ethnicity	
Caucasian	126 (6.73 %)
Chinese	1008 (54.60 %)
Filipino	132 (7.15 %)
Indonesian	102 (5.53 %)
Thai	255 (13.81 %)
Malay	98 (5.31 %)
Vietnamese/Cambodian	24 (1.30 %)
Indian/Sri Lankan	64 (3.47 %)
Other^a^	37 (2.00 %)
Female gender	1723 (93.34 %)
Age at diagnosis (years)	29.34 (12.35)
Age at diagnosis ≤30 years	973 (52.71 %)
Disease duration at enrollment (years)	8.64 (8.50)
Disease duration at enrollment ≤1 year	149 (8.07 %)
Current smoker	67 (3.63 %)
First-degree relative with SLE	117 (6.34 %)
Highest attained education level	
Primary	242 (13.11 %)
Secondary	572 (30.99 %)
Tertiary	618 (33.48 %)
ACR criteria^b^	
Malar rash	1087 (58.88 %)
Discoid rash	290 (15.71 %)
Photosensitivity	537 (29.09 %)
Mouth ulcers	670 (36.29 %)
Arthritis	1205 (65.28 %)
Serositis	313 (16.96 %)
Renal	803 (43.50 %)
Neurologic	160 (8.67 %)
Haematologic	1118 (60.56 %)
Immunologic	1547 (83.80 %)
ANA	1627 (88.14 %)
Number of ACR criteria for SLE	5.07 (1.39)
Number of SLICC criteria for SLE	5.70 (2.47)
SLICC-DI score at enrollment	0 (0–1)
Damage present at enrollment^d^	694 (37.59 %)
PGA at enrollment	0.6 (0.3–1)
Mild flare since last clinical review	210 (11.38 %)
Severe flare since last clinical review	111 (5.94 %)
SLEDAI-2 K score	4 (2–6)
SLEDAI-2 K no complement or dsDNA	0 (0–4)

^a^Other includes Hispanic, African, other South-East Asian, Pacific Islander and mixed ethnicity. ^b^Ever present arthritis (two or more joints with tenderness, swelling or effusion), serositis (pleuritis or pericarditis), renal disorder (persistent proteinuria >0.5 g/day, or presence of cellular casts), neurologic disorder (seizures or psychosis not attributable to other causes), haematologic disorder (haemolytic anaemia, leukopenia, lymphopenia or thrombocytopenia), immunologic criteria (presence of anti-dsDNA antibody, anti-Sm antibody, or positive finding of antiphospholipid antibodies). ^c^Percent present shown in table, percent absent and missing not shown in table. ^d^SLICC-DI >0. *SLE* systemic lupus erythematosus, *ACR* American College of Rheumatology, *SLEDAI* SLE disease activity index, *SLICC* Systemic Lupus International Collaborating Clinics, *DI* damage index, *PGA* Physician Global Assessment, *ANA* antinuclear antibody, *dsDNA* double-stranded DNA

Pooled data from all sites were analysed using STATA v13 (StataCorp, College Station, TX, USA). Data are reported as mean (standard deviation (SD)) for normally distributed continuous variables and median (interquartile range (IQR)) for skewed continuous data. The chi-squared test was used for categorical comparisons. Univariate simple logistic regression was used to identify predictors of LLDAS. Variables with *p* value ≤0.2 in simple logistic regression analysis were then checked for confounding and multicollinearity, prior to inclusion in stepwise multivariable logistic regression analysis for LLDAS. Model properties including sensitivity and specificity, receiver operating characteristic (ROC) and *p* value for the Hosmer-Lemeshaw test for goodness of fit are available in Additional file 1: Table S1.

## Results

### Demographics and disease characteristics

A total of 1846 patients were recruited. In this cohort, 93 % of patients were female, with a mean age at diagnosis of 29 (SD ± 12.4) years and mean disease duration of 8.6 (SD ± 8.5) years at the time of recruitment. There were 149 patients (8 %) recruited within 12 months of disease diagnosis. More than 50 % of patients were of Chinese ethnicity, 7 % of patients were Caucasian, and the remainder represented other ethnic groups native to the region (Table 1). Other baseline demographics are presented in Table 1.

Disease manifestations were determined from the ACR criteria on an “ever present” basis (Table 1). More than half of the patients had a history of malar rash, arthritis and haematologic and immunologic manifestations, and 803 patients (44 %) had a history of renal disease. The median SLEDAI-2 K at enrollment was 4 (IQR 2–6) (Table 1). There were 694 patients (38 %) had irreversible damage at recruitment (SLICC-DI >0), and the median SLICC-DI score was 0 (IQR 0–1). In total, 1430 patients (77.5 %) were on prednisolone, with a mean dose of 11 mg (SD ± 12.8 mg) per day (Table 2).

**Table 2:** Medication taken at enrollment

Medication	Number (%)	Mean dose (SD)	Dose range
Prednisolone	1430 (77.46 %)	11.08 mg (12.78)	0.50–200 mg
Antimalarial	1333 (72.21 %)	291.19 mg (104.56)^a^	28.57–600 mg^a^
Methotrexate	75 (4.06 %)	13.79 mg (6.73)	2.50–50 mg
Azathioprine	412 (22.32 %)	73.99 mg (30.29)	12.50–200 mg
Mycophenolate mofetil	306 (16.58 %)	1247.70 mg (546.96)	50–3000 mg
Mycophenolic acid	41 (2.22 %)	1102.93 mg (645.86)	180–2160 mg
Leflunomide	38 (2.06 %)	15.53 mg (5.49)	10–30 mg
Cyclosporine	35 (1.90 %)	126.43 mg (65.29)	50–300 mg
Cyclophosphomide^b^	73 (3.95 %)	N/A	N/A
Rituximab^b^	13 (0.70 %)	N/﻿A	N/A
Belimumab^b^	15 (0.81 %)	N/﻿A	N/A
Any Immunosuppressant^c^	940 (50.92 %)	N/A	N/A

^a^Based on hydroxychloroquine dosing - Indonesia and Thailand predominantly use chloroquine. ^b^Taken in the last 6 months. ^c^Either methotrexate, azathioprine, mycophenolate, leflunomide, cyclosporine, cyclophosphamide, rituximab and/or belimumab. Maximum recommended dose: hydroxychloroquine ≤400 mg; methotrexate ≤30 mg; azathioprine ≤200 mg; mycophenolate mofetil ≤3000 mg; mycophenolic acid ≤2160 mg; leflunomide ≤20 mg

N/A - dosing not applicable

### Frequency of meeting criteria for LLDAS

All of the patients fulfilled at least one criterion of LLDAS (Table 3). The most frequently present criterion (*n* = 1838 patients (99.6 %)) was the criterion relating to immunosuppressive medications, with only eight patients exceeding a maximum recommended dose. The least frequently present criterion (1171 patients (63.4 %)) was SLEDAI-2 K ≤4 without activity in a major organ system, followed by the glucocorticoid dose criterion (68.2 %). A higher proportion of patients achieved PGA ≤1 than achieved SLEDAI ≤4 (76 % vs. 63 %, *p* < 0.001). Despite a high frequency of attainment of individual criteria, only 810 patients (43.9 %) fulfilled all five criteria for LLDAS.

**Table 3:** Lupus low disease activity state (LLDAS) frequency

Descriptors of disease activity	Number (%) (*n* = 1846)
1. SLEDAI-2 K ≤4, with no activity in major organ systems (renal, CNS, cardiopulmonary, vasculitis, haemolytic anaemia, fever) and no gastrointestinal activity	1171 (63.43 %)
2. No new features of lupus disease activity compared to the previous assessment	1574^a^ (85.27 %)
3. SELENA-SLEDAI Physician Global Assessment (PGA, scale 0–3) ≤1	1400 (75.84 %)
Immunosuppressive medications	
4. Current prednisolone (or equivalent) dose ≤7.5 mg daily	1258 (68.15 %)
5. Well-tolerated standard maintenance doses of immunosuppressive drugs and approved biologic agents, excluding investigational drugs^b^	1838 (99.57 %)
All 5 criteria present	810 (43.88 %)

^a^Based on flares (see “Methods”). ^b^Calculated as not exceeding maximum recommended dose: hydroxychloroquine ≤400 mg; methotrexate ≤30 mg; azathioprine ≤200 mg; mycophenolate mofetil ≤3000 mg; mycophenolic acid ≤2160 mg; leflunomide ≤20 mg. *SLE* systemic lupus erythematosus, *SELENA* Safety of Estrogens in Lupus Erythematosus National Assessment trial, *SLEDAI* SLE disease activity index, *CNS* central nervous system, *PGA* Physician Global Assessment

### Determinants of presence of LLDAS

Multiple independent variables had a significant association with LLDAS in univariate analysis (Table 4). Younger age at diagnosis (OR 0.77, 95 % CI 0.64–0.93, *p* = 0.006) and shorter disease duration (OR 0.34, 95 % CI 0.23–0.51, *p* < 0.001) were negatively associated with LLDAS. A history of discoid rash (OR 0.73, 95 % CI 0.57–0.95, *p* = 0.02) or renal disease (OR 0.63, 95 % CI 0.53–0.77, *p* < 0.001), or current anti-dsDNA positivity (OR 0.55, 95 % CI 0.46–0.68, *p* < 0.001) and hypocomplementaemia (low C3 and or C4; OR 0.45, 95 % CI 0.37–0.55, *p* < 0.001) were all negatively associated with LLDAS. No significant differences were observed in ethnicity, gender or educational level. In multivariable logistic regression analysis, variables that remained significantly negatively associated with LLDAS included disease duration ≤1 year (OR 0.31, 95 % CI 0.19–0.49, *p* < 0.001), history of discoid rash (OR 0.66, 95 % CI 0.49–0.89, *p* = 0.006) or renal disease (OR 0.60, 95 % CI 0.48–0.75, *p* < 0.001); and current elevated anti-dsDNA (OR 0.65, 95 % CI 0.53–0.81, *p* < 0.001) or hypocomplementaemia (OR 0.52, 95 % CI 0.40 − 0.67, *p* < 0.001). Patients from countries with a high GDP (PPP) per capita were significantly more likely to be in LLDAS than patients from countries with a lower GDP (PPP) per capita (OR 1.57, 95 % CI 1.25–1.98, *p* < 0.001). Model properties for the aforementioned variables are presented in Additional file 1: Table S1.

**Table 4:** Determinants of lupus low disease activity state (LLDAS)

Independent variable	Number (%) in LLDAS	Univariable logistic regression for LLDAS		Multivariable logistic regression for LLDAS	
		OR (95 % CI)	*p*	OR (95 % CI)	*p*
Ethnicity^a^					
Caucasian	63 (50.00)	Reference		Reference	
Asian	700 (43.24)	0.76 (0.53–1.09)	0.14	1.23 (0.82–1.86)	0.31
Gender					
Female	758 (43.99)	Reference		N/A	
Male	52 (42.28)	0.93 (0.64–1.35)	0.71		
Education					
Primary	113 (46.69)	Reference		N/A	
Secondary	229 (40.03)	0.76 (0.56–1.03)	0.21		
Tertiary	254 (41.10)	0.80 (0.59–1.07)	0.30		
Age at diagnosis					
>30 years	407 (47.22)	Reference		Reference	
≤30 years	397 (40.80)	0.77 (0.64–0.93)	0.006	0.86 (0.70–1.06)	0.16
Disease duration					
>1 year	765 (46.48)	Reference		Reference	
≤1 year	34 (22.82)	0.34 (0.23–0.51)	<0.001	0.31 (0.19–0.49)	<0.001
Clinical features^b^					
Malar rash					
No	331 (43.61)	Reference		N/﻿A	
Yes	479 (44.07)	1.02 (0.82–1.23)	0.85		
Discoid rash					
No	701 (45.05)	Reference		Reference	
Yes	109 (37.59)	0.73 (0.57–0.95)	0.02	0.66 (0.49–0.89)	0.006
Photosensitive					
No	562 (42.93)	Reference		Reference	
Yes	248 (46.18)	1.14 (0.93–1.40)	0.20	1.18 (0.93–1.50)	0.16
Mouth Ulcers					
No	527 (44.81)	Reference		N/A	
Yes	283 (42.24)	0.90 (0.74–1.09)	0.28		
Arthritis					
No	263 (41.03)	Reference		Reference	
Yes	547 (45.39)	1.19 (0.98–1.45)	0.07	0.98 (0.78–1.23)	0.87
Serositis					
No	673 (43.90)	Reference		N/A	
Yes	137 (43.77)	0.99 (0.78–1.27)	0.97		
Renal					
No	508 (48.71)	Reference		Reference	
Yes	302 (37.61)	0.63 (0.53–0.77)	<0.001	0.60 (0.48–0.75)	<0.001
Neurologic					
No	732 (43.42)	Reference		Reference	
Yes	78 (48.75)	1.24 (0.90–1.72)	0.20	1.31 (0.90–1.91)	0.16
Haematologic					
No	289 (39.70)	Reference		Reference	
Yes	521 (46.60)	0.22 (0.03–1.76)	0.09	1.21 (0.97–1.51)	0.09
Immunologic					
No	145 (48.59)	Reference		N/A	
Yes	665 (42.99)	0.80 (0.63–1.03)	0.08		
ANA					
No	101 (46.12)	Reference		N/A	
Yes	709 (43.58)	0.90 (0.68 − 1.20)	0.48		
Baseline damage					
SLICC-DI = 0	505 (44.84)	Reference		N/A	
SLICC-DI >0	308 (42.95)	1.00 (0.83–1.21)	0.96		
Current high anti-dsDNA					
No	434 (51.12)	Reference		Reference	
Yes	341 (36.43)	0.55 (0.46–0.68)	<0.001	0.65 (0.53–0.81)	<0.001
Current low complement					
No	636 (49.88)	Reference		Reference	
Yes	142 (29.83)	0.45 (0.37–0.55)	<0.001	0.52 (0.40–0.67)	<0.001
GDP ≤ $25,000^c^	324 (36.00)	Reference		Reference	
GDP > $25,000	486 (51.37)	1.89 (1.56–2.26)	<0.001	1.57 (1.25–1.98)	<0.001

Multivariable model choice - variables with a *p* value ≤0.20 or clinical association in univariable regression were tested for multicolliniarity and confounding. Final variable list - GDP (PPP) per capita, ethnicity, age at diagnosis, disease duration, discoid rash, photosensitivity, arthritis, renal disease, neurologic disease, haematologic disease, dsDNA and complement. ^a^Ethnicity – no significant differences were seen between Asian ethnicity subgroups. ^b^Ever present, arthritis (two or more joints with tenderness, swelling or effusion), serositis (pleuritis or pericarditis), renal disorder (persistent proteinuria >0.5 g/day, or presence of cellular casts), neurologic disorder (seizures or psychosis not attributable to other causes), haematologic disorder (haemolytic anaemia, leukopenia, lymphopenia or thrombocytopenia), immunologic criteria (presence of anti-dsDNA antibody, anti-Sm antibody, or positive finding of antiphospholipid antibodies). ^c^Countries with GPD < $25,000 - China, Indonesia, Malaysia, Philippines, Thailand; countries with GDP > $25,000 - Australia, Hong Kong, Singapore, Taiwan. GDP measured in US dollars. *N/A* variable not included in multivariable regression model

*LLDAS* lupus low disease activity state, *SLICC* Systemic Lupus International Collaborating Clinics, *DI* damage index, *dsDNA* double-stranded DNA, *ANA* antinuclear antibody, *GDP* gross domestic product

Analysis of the effect of disease manifestations as defined by ACR criteria [12] on individual LLDAS criteria (Additional file 1: Table S2) revealed that patients with immunologic manifestations were less likely to have SLEDAI-2 K ≤4 (OR 0.73, 95 % CI 0.56–0.96, *p* = 0.02). A history of renal disease was significantly associated with lower odds of meeting any of the individual LLDAS criteria. The presence of damage (SLICC-DI >0) at recruitment was significantly associated with lower frequency of meeting several LLDAS criteria including SLEDAI ≤4 (OR 0.79, 95 % CI 0.65–0.96, *p* = 0.02), absence of flare (OR 0.67, 95 % CI 0.52–0.88, *p* = 0.003) and PGA ≤1 (OR 0.64, 95 % CI 0.51–0.79, *p* < 0.001).

## Discussion

The authors have commenced a large prospective longitudinal study to validate the recently reported definition of LLDAS as being predictive of protection from damage accrual in SLE [11]. In the current cross-sectional study of data collected at recruitment into this large multinational cohort, we have shown that 44 % of patients with SLE met LLDAS criteria for low disease activity at a single point in time. This is the first multinational study to focus on the recent definition of LLDAS, and the frequency of LLDAS observed closely matches the 41 % frequency of LLDAS attainment in our initial retrospective single-centre validation study [11]. If LLDAS attainment or maintenance is shown to translate into improved patient outcomes, such as is the case for attainment of minimal disease activity in RA [9], this frequency of attainment, especially compared to more stringent cutoffs such as remission, suggests that LLDAS could represent a treatment target to use in SLE strategy trials and in clinical trials of novel therapies. Conversely, the fact that the majority of patients did not meet criteria for LLDAS speaks to the inadequate state of current treatment of SLE.

The definition of LLDAS [11] incorporates cutoffs for both disease activity and treatment burden. It refers to a desired clinical state, rather than a treatment response or change in disease activity, therefore representing a tool with which to stratify clinically diverse disease manifestations in a binary fashion, i.e. a patient is either in LLDAS or not. LLDAS was designed to take into account validated measures of disease activity [20] and treatment variables, in view of the fact that treatment, especially with glucocorticoids, is known to contribute to poor long-term outcomes in SLE [21, 22]. In the current study, the second most frequent reason for not attaining LLDAS was glucocorticoid dose >7.5 mg/day. Although it is clear that higher disease activity over time is associated with worse outcomes [23], measures of disease activity alone, such as the SLEDAI-2 K or the British Isle Lupus Assessment Group (BILAG) measure, do not take into account treatment burden and therefore omit consideration of a major contributor to long-term harm in SLE. Similarly, measures of treatment response such as the SLE Responder Index [24], although they combine different measures of disease activity, do not represent a target state and do not include treatment variables.

Our finding that 99 % of patients met at least one LLDAS criterion, but only 44 % of patients met all five criteria, supports the value of including multiple variables in the definition of LLDAS. A higher proportion of patients achieved PGA ≤1 than achieved SLEDAI ≤4, potentially because of the inclusion of serological and clinical activity in the SLEDAI-2 K; the presence of dsDNA antibodies and hypocomplementaemia equates to 4 points on the SLEDAI-2 K, therefore any additional manifestation will result in the patient exceeding the SLEDAI-2 K cutoff for LLDAS.

The size of this cohort allowed us to evaluate factors associated with the presence of LLDAS. Some of the most common clinical manifestations of active disease in SLE are immunologic, cutaneous and renal disease [3], each of which was significantly negatively associated with LLDAS in multivariable regression. Disease duration of less than one year was also negatively associated with LLDAS, consistent with the observation that newly diagnosed patients are more likely to have active disease [18].

Our study has shed some further light on treatment practices in tertiary lupus centres. The lower frequency of use of immunosuppressants in this cohort may be related to issues with access to or availability of medications in some Asian countries, which has been previously described [26]; certainly in our recent single centre report based on an Australian cohort, the frequency of immunosuppressant use was considerably higher than in the present study [11]. The mean daily dose of prednisolone of 11 mg/day is higher than doses reported in recent studies in single-centre cohorts with similar mean disease duration [11, 22]. As prolonged prednisolone use is known to contribute to significant morbidity in SLE [27], the consequences of high glucocorticoid dosing in this cohort with mean disease duration at recruitment close to 9 years will need to be further assessed.

It is well-established that personal socioeconomic status contributes to disease activity [28] and disease damage [29] in SLE. A recent study from the Asia Pacific region has also shown that national social wealth and development has a very strong association with 5-year survival among patients with SLE [25]. As such, we believed it important to include an index of socioeconomic wealth in analyzing predictors of LLDAS. Indeed, in our study, patients from countries with higher GDP per capita (PPP) were significantly more likely to meet all criteria for LLDAS. The GDP (PPP) per capita is adjusted for the cost of living and is therefore useful for comparing standards of living rather than just national wealth [19]. The main drawback of this measure is that it does not measure personal socioeconomic status, which would also vary from patient to patient. However, education level, a potential surrogate marker of individual socioeconomic standing, was not predictive of LLDAS.

Certain limitations apply to the current study. Because of the cross sectional nature of the current analysis, we are unable to ascertain whether time spent in LLDAS is associated with less damage accrual, as was shown in the original retrospective single-centre validation of LLDAS [11]. The cohort described here is the subject of a longitudinal study intended to determine the association of LLDAS attainment with outcomes including damage accrual. Additionally, the published definition of LLDAS requires the absence of new disease manifestations, which is not possible to measure in a cross-sectional study; we replaced this with a requirement for the absence of flare as measured using SFI, which is likely to have been more rather than less stringent. In addition, identification of the “well-tolerated immunosuppressive” component of LLDAS was modified due to the inability to determine dose change or tolerance at recruitment. This resulted in a high proportion of patients fulfilling this criterion, and use of the original definition in our longitudinal study may result in a lower overall frequency of LLDAS.

## Conclusions

In conclusion, a validated definition of low disease activity has transformed both clinical care and clinical trial design in RA. Defining a treatment outcome that is attainable in an achievable proportion of patients and associated with improved long-term outcomes is something that has eluded SLE researchers until recently. Here, we have shown in a large multi-national and multi-ethnic cohort that LLDAS is attainable in a significant proportion of patients analysed at a single point in time, suggesting this definition is practical for use in long-term studies. We have also identified clinical variables associated with reduced likelihood of LLDAS, which if confirmed in longitudinal studies, may help with early identification of patients at higher risk. The next step in validation of LLDAS as an outcome measure in SLE is the definitive evaluation of whether LLDAS attainment or maintenance is associated with protection from long-term adverse outcomes such as damage accrual. This validation study, which will also allow for potential refinement of the LLDAS definition based on identifying variables that are most predictive of good outcomes, as was done for the recently described re-definition of remission in RA [30], is underway. That less than half of patients studied met the definition of LLDAS serves to underline the need for advances in the care of SLE, for which new strategies and new drugs are needed.

## Additional file

Additional file 1: Table S1. Multiple logistic regression model properties. **Table S2.** Effect of disease manifestations and damage at recruitment on LLDAS components. (DOCX 59 kb)

## Abbreviations

ACR: American College of Rheumatology
ANA: antinuclear antibody
APLC: Asia Pacific Lupus Collaboration
CNS: central nervous system
DI: Damage Index
dsDNA: double-stranded DNA
GDP: gross domestic product
LLDAS: lupus low disease activity state
PGA: Physician Global Assessment
RA: rheumatoid arthritis
ROC: receiver operating characteristic
SELENA: Safety of Estrogens in Lupus Erythematosus National Assessment
SFI: systemic lupus erythematosus flare index
SLE: systemic lupus erythematosus
SLEDAI: Systemic Lupus Erythematosus Disease Activity Index
SLICC: Systemic Lupus International Collaborating Clinics
