# Supplementary material for: Frequency and predictors of the lupus low disease activity state in a multi-national and multi-ethnic cohort
Source: Arthritis Res Ther. 2016 Nov 9;18:260. doi: 10.1186/s13075-016-1163-2 (PMC5103412; doi:10.1186/s13075-016-1163-2)
Supplement: Additional file 1: Table S1. — Multiple logistic regression model properties. Table S2. Effect of disease manifestations and damage at recruitment on LLDAS components. (DOCX 59 kb) [file 13075_2016_1163_MOESM1_ESM.docx]

**Supplementary tables**

**Supplementary Table 1: Multiple logistic regression model properties.**

| Sensitivity | 50.1% (95%CI: 46.4%-53.9%) |
| --- | --- |
| Specificity | 73.4% (95% CI: 70.3%-76.3%) |
| Hosmer-Lemeshaw goodness of fit | *p*=0.19* |
| Area under the ROC curve | 0.67 (95% CI: 0.64-0.70)^ |

*H-L test p>0.05 indicates the model fits the data well i.e. observed and predicted outcome are not significantly different

^Higher area under ROC curve indicates high predictive accuracy

**Supplementary Table 2: Effect of disease manifestations and damage at recruitment on LLDAS components***

| Independent variable | SLEDAI-2K≤4 and no organ activity | | No new activity (flares only) | | PGA≤1 | | Prednisolone  ≤7.5mg | | All 5 LLDAS criteria | |
| --- | --- | --- | --- | --- | --- | --- | --- | --- | --- | --- |
| Disease manifestation^#^  Malar rash  Discoid rash  Photosensitivity  Mouth Ulcers  Arthritis  Serositis  Renal  Neurologic  Haematologic  Immunologic  Positive ANA | OR  95% CI  0.98  0.80-1.18  0.81  0.62-1.06  1.05  0.85-1.30  0.87  0.71-1.06  0.94  0.77-1.15  0.94  0.73-1.21  **0.53**  **0.44-0.65**  1.01  0.72-1.42  1.20  0.99-1.46  **0.73**  **0.56-0.96**  0.98  0.73-1.31 | *p*  0.80  0.11  0.64  0.16  0.58  0.65  **<0.001**  0.93  0.06  **0.02**  0.87 | OR  95% CI  0.82  0.63-1.07  0.92  0.65-1.30  0.76  0.58-1.00  **0.59**  **0.46-0.77**  0.86  0.65-1.13  0.81  0.58-1.13  **0.76**  **0.59-0.98**  1.08  0.68-1.73  0.84  0.64-1.10  0.83  0.57-1.19  **0.56**  **0.34-0.90** | *p*  0.14  0.63  0.06  **<0.001**  0.28  0.22  **0.04**  0.74  0.21  0.31  **0.01** | OR  95% CI  1.10  0.88-1.36  0.88  0.66-1.17  0.98  0.78-1.24  1.00  0.80-1.24  0.93  0.74-1.16  1.15  0.86-1.54  **0.69**  **0.56-0.86**  0.99  0.67-1.44  1.18  0.96-1.47  0.81  0.60-1.10  1.18  0.86-1.63 | *p*  0.40  0.38  0.88  0.99  0.45  0.33  **0.001**  0.95  0.12  0.17  0.31 | OR  95% CI  0.91  0.75-1.12  1.05  0.80-1.37  1.05  0.85-1.30  0.91  0.74-1.12  **1.43**  **1.17-1.75**  0.96  0.74-1.24  **0.70**  **0.58-0.85**  1.37  0.96-2.01  **1.28**  **1.05-1.57**  0.86  0.65-1.24  **0.62**  **0.44-0.86** | *p*  0.37  0.74  0.66  0.37  **0.001**  0.76  **0.001**  0.07  **0.014**  0.26  **0.003** | OR  95% CI  1.02  0.85-1.23  **0.73**  **0.57-0.95**  1.14  0.93-1.40  0.90  0.74-1.09  1.19  0.98-1.45  0.99  0.78-1.27  **0.63**  **0.53-0.77**  1.24  0.90-1.72  0.22  0.03-1.76  0.80  0.63-1.03  0.90  0.68-1.20 | *p*  0.85  **0.02**  0.20  0.28  0.07  0.97  **<0.001**  0.20  0.09  0.08  0.48 |
| Damage present at baseline  (SLICC-DI >0) | **0.79**  **0.65-0.96** | **0.02** | **0.67**  **0.52-0.88** | **0.003** | **0.64**  **0.51-0.79** | **<0.001** | 1.27  0.99-1.56 | 0.06 | 0.94  0.78-1.13 | 0.52 |

*No significant associations with Medication not exceeding maximum recommended dose (not shown in table).

^#^Ever present. Arthritis (two or more joints with tenderness, swelling or effusion), serositis (pleuritis or pericarditis), renal disorder (persistent proteinuria >0.5grams per day, or presence of cellular casts), neurologic disorder (seizures or psychosis not attributable to other causes), haematologic disorder (haemolytic anaemia, leukopaenia, lymphopaenia or thrombocytopaenia), immunologic criteria (presence of anti-dsDNA antibody, anti-Sm antibody, or positive finding of antiphospholipid antibodies).

Abbreviations: LLDAS (Lupus Low Disease Activity State); SLE (systemic lupus erythematosus); SLEDAI (SLE disease activity index); PGA (physician global assessment); SLICC (Systemic Lupus International Collaborating Clinics); DI (damage index); dsDNA (double stranded DNA); ANA (antinuclear antibody)
